# The risk of new vertebral fracture after percutaneous vertebral augmentation in patients suffering from single-level osteoporotic vertebral compression fractures: A meta-analysis and systematic review

**DOI:** 10.1097/MD.0000000000035749

**Published:** 2023-11-17

**Authors:** Zhaoyang Qiu, Peng Wang, Yuqiang Chao, Yang Yu

**Affiliations:** a Panjin Central Hospital, Panjin City, China; b The First Affiliated Hospital of Jinzhou Medical University, Jinzhou, China.

**Keywords:** conservative treatment, kyphoplasty, new vertebral fracture, osteoporotic vertebral compression fracture, percutaneous vertebral augmentation, vertebroplasty

## Abstract

**Background:**

To investigate the effect of Vertebral augmentation (VA) in the treatment of single-level osteoporotic vertebral compression fractures (OVCFs) on new vertebral fractures.

**Methods:**

Electronic databases Pubmed, Embase, and the Cochrane Central Register of Controlled Trials were searched from database creation to 5 September 2022. Eligible studies had to use VA as an intervention and conservative treatment as a control group. Studies had to explicitly report whether new vertebral fractures occurred during follow-up. Data were extracted by multiple investigators. Data were pooled using random or fixed effects models depending on the degree of heterogeneity.

**Results:**

Of the 682 articles screened, 7 met the inclusion criteria and were included in the analysis, giving a total of 1240 patients. Meta-analysis showed that VA (OR = 2.10, 95% CI: 1.35–3.28, *P* = .001) increased the risk of new postoperative vertebral fractures compared with conservative treatment. Subgroup analyses showed that the risk was greater in the group with a follow-up time greater than 1 year (OR = 2.57, 95% CI: 1.06–6.26, *P* = .001). Compared with conservative treatment, VA (OR = 2.17, 95% CI: 1.23–3.82, *P* = .007) increased the risk of postoperative adjacent vertebral fracture.

**Conclusion subsections:**

VA is associated with an increased risk of new vertebral fractures and adjacent vertebral fractures following single-level OVCFs. With longer follow-ups, new vertebral fractures may be more significant. Clinical surgeons should pay attention to long-term postoperative complications and choose treatment carefully.

## 1. Introduction

Osteoporosis has already been one of the chronic diseases afflicting the elderly.^[1]^ Osteoporotic vertebral compression fractures (OVCFs) are the most common fragility fractures among osteoporotic fractures.^[2]^ Percutaneous vertebroplasty (PV) and Percutaneous kyphoplasty (PK), which are common in percutaneous vertebral augmentation (VA), have been widely used in the treatment of OVCFs. Many studies have now shown that PV/PK can effectively relieve pain caused by OVCFs.^[3–11]^ However, most scholars seem to pay too much attention to postoperative Visual Analogue Scale (VAS) changes, and there are few reports on the impact of new postoperative vertebral fractures, and this issue is controversial. Some scholars believe that there is no increased risk.^[9,12–17]^ Because osteoporosis is a degenerative disease, bone mineral density will gradually decrease with age, new vertebral fractures are because the patient’s own vertebral bone mineral density is low, and new vertebral fractures are only a natural course, inevitable, and unrelated to PA. However, some scholars hold the opposite view.^[18–22]^ Because bone cement injected during surgery leads to increased vertebral bone stiffness, changes in vertebral body height can lead to changes in spinal stress, as well as some other factors that are not yet clear. These may all be contributing factors to new vertebral fractures following VA treatment. Most of the patients encountered by surgeons in clinical practice had OVCFs involving only a single vertebral body, but most studies and meta-analyses did not investigate single-level OVCFs and multilevel OVCFs separately. The effect of multilevel OVCFs on new vertebral fractures may be much greater than that of single-level OVCFs, and pooling the data between the two may affect the results of single-level OVCFs. This may also be the reason why this issue is more controversial. Therefore, we aimed to investigate new vertebral fractures after VA surgery for single-level OVCFs and provide some help for clinical surgeons’ treatment decisions.

## 2. Methods

This systematic review and meta-analysis applies and adheres to the Preferred Reporting Items for Systematic Reviews and Meta-Analyses (PRISMA) reporting guidelines and closely follows the Cochrane Handbook for Systematic Reviews.

### 2.1. Search strategy

Our study was performed according to the Preferred Reporting Items Guidelines for Systematic Reviews and Meta-Analyses (PRISMA), and we searched PubMed, Embase, and the Cochrane Library for eligible studies published until September 5, 2022. Because the number of high-quality randomized controlled trials was small, we did not limit the study design. However, we used conservative treatment as a control group, which can effectively reduce heterogeneity. Subject terms we searched included Spinal Fractures, Osteoporosis, Osteoporotic Fractures, Compression fractures, Vertebroplasty, Kyphoplasty, Treatment Outcome, Placebo, and Conservative Treatment Outcome. The subject words of these words and their free words are retrieved, and a retrieval formula is constructed using Boolean operators. At the same time, we read the meta-analysis and review in the past 5 years and selected studies from the included studies and references. We also searched the references of all included studies as well as relevant reviews, and no language and publication country restrictions were imposed. The full search formula can be found in the Appendix, Supplemental Digital Content, http://links.lww.com/MD/K692. Duplicate search results removed. The study protocol has been registered in PROSPERO (CRD42022368858).

### 2.2. Eligibility criteria

The included studies had to report that patients’ OVCFs were single-level vertebral bodies. Report 20 or more specimens per group. PV/PK was used as an intervention, and conservative treatment or sham operation was used as a control group. New vertebral fractures occurring during the follow-up period were reported and the follow-up time was greater than or equal to 3 months. Studies such as reviews, meta-analyses, case reports, letters to editors, conference papers, expert consensus, guidelines, finite element analysis, cadaveric studies, and animal experiments were excluded. Pathologic fractures, burst fractures, kummell’s disease, studies in which non-VA was the treatment regimen, and studies in which patients with multilevel OVCFs and single-level OVCFs were not analyzed separately were also excluded. We did not restrict the language of included studies and the country of publication.

### 2.3. Information sources

On 5 September 2022, we searched PubMed, Embase, and the Cochrane Library for eligible studies published from database establishment until 5 September 2022. At the same time, we read the meta-analysis and review in the past 5 years and selected studies from the included studies and references.

### 2.4. Study selection and data extraction

Two reviewers performed study screening and data extraction of abstracts and full texts according to the inclusion criteria. Any conflicts during this period were decided by discussion or by a third-party reviewer. Data were extracted into Excel following guidance provided by Cochrane Handbook for Systematic Reviews. The variables extracted included authors, publication year, study design, study start, and end dates, geographic location, follow-up time, baseline characteristics of the included sample, number of patients in the VA and CT groups, number of new vertebral fracture events during follow-up, and whether any patients received osteoporosis treatment during follow-up.

### 2.5. Risk of bias and study quality

The Cochrane Risk of Bias 2 tool was used to assess the risk of bias and study quality of randomized controlled trial studies. The risk of bias in randomized controlled trials could be described as “low risk,” “some concerns,” and “high risk.” Newcastle-Ottawa Scale was used to assess the risk of bias and study quality of cohort studies. Cohort studies assessed total scores up to 9. A total score of 7 or more points indicates high study quality. Two reviewers assessed the risk of bias and study quality alone, and any conflicts were resolved by discussion or by a third-party reviewer.

### 2.6. Selection process

**Figure 1.**
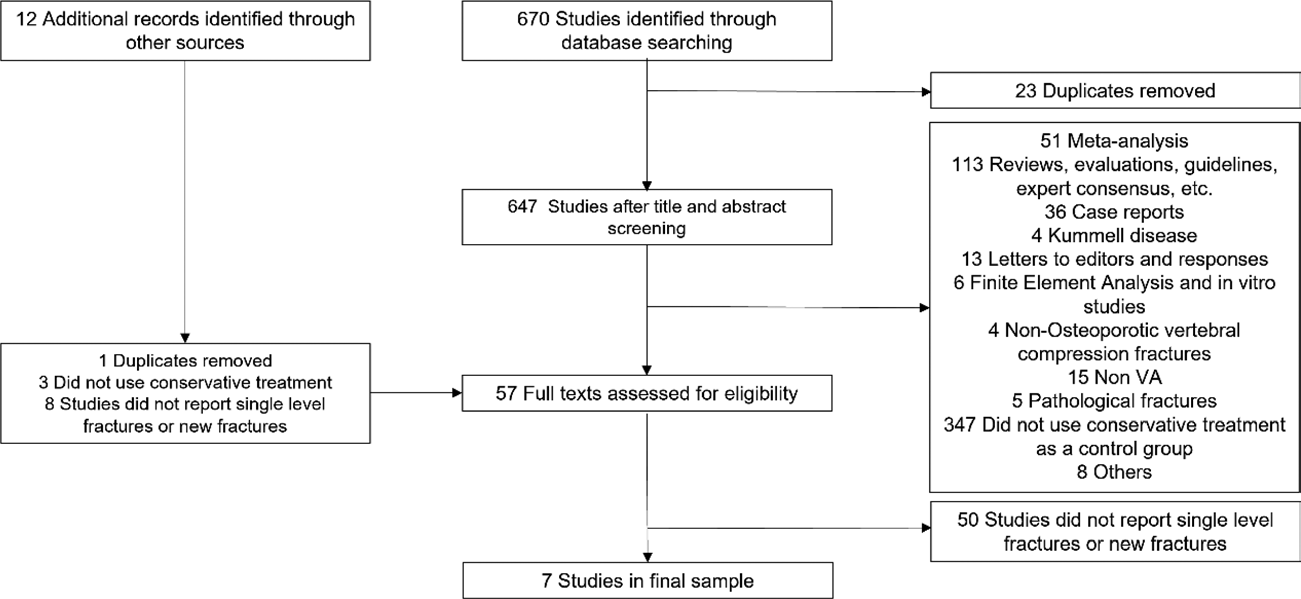
Flowchart of the selection of included studies.

The screening was performed according to the inclusion criteria and the screening process was performed independently by 2 reviewers, any conflicts were resolved by discussion or by a third-party reviewer and the screening flowchart is shown in Figure 1.

### 2.7. Statistical analysis

The statistical software we used was Review manager 5.4. Because it is a dichotomous variable, we chose to use the Mantel-Haenszel test in statistical methods. For effect measures, we selected Odds Ratio (OR). We performed heterogeneity analysis, using *I*^2^ and *P* values to describe heterogeneity, and we used the fixed effects model when heterogeneity analysis *I*^2^ was < 50%, otherwise random effects model was used. 95% was chosen for study confidence intervals and overall confidence intervals. We combined effect sizes and calculated *P* values when *P* values for heterogeneity analysis were > .05. We made forest plots and funnel plots because we included only 7 studies, and it was not necessary to assess publication excursions by observing if the funnel plots were symmetrical. We also performed a meta-analysis for adjacent vertebral fractures using the same methodology. We performed a subgroup meta-analysis of the study design, follow-up time, and whether any patients received osteoporosis treatment during follow-up. In terms of sensitivity analysis, we sequentially excluded one study to observe heterogeneity and whether there were significant changes in the results. We performed sensitivity analysis on STATA 15 software and generated analysis plots.

## 3. Results

### 3.1. Study selection

We retrieved a total of 670 studies and excluded 23 duplicates. Through titles and abstracts, we excluded 51 meta-analyses, 113 reviews, evaluations, guidelines, and expert consensus, 36 case reports, 8 significantly unrelated, 4 kummell's disease, 13 letters and replies to editors, 6 finite element analysis and in vitro studies, 4 non-OVCFs, 15 non-VA, 5 pathological fractures, and 347 studies did not use conservative treatment as a control group, and a total of 602 were excluded. After full-text reading of 45 studies, 38 studies that did not report single-level OVCFs and new vertebral fractures that occurred during follow-up were excluded, and a total of 7 studies were included in the data analysis. We read the meta-analysis and review in the past 5 years, read the titles and abstracts from the included studies and references, and preliminarily selected 12 studies that met the criteria. However, after reading the full text, no article met the inclusion criteria. See Figure 1 for details.

### 3.2. Study characteristics

Of the 7 included studies, 2 were randomized controlled trials and 5 were cohort studies. Information on baseline characteristics of subgroups and study participants is detailed in Table 1.

**Table 1:** Characteristics of the eligible study in this meta-analysis.

Author	Year	Region	Study design	Age	All patients	Male/female	Follow-up (mo)	Osteoporosis therapy	NOS or ROB2
Bae	2019	America, Korea	Cohort study	75.1 ± 10.8	164	43/121	12	Yes	8
Chen	2015	China	RCT	66.57 ± 8.52	84	37/47	12–54	No	Low
Ee	2015	Singapore	Cohort study	75.99 ± 8.91	363	56/307	24	No	7
Kim	2016	Korea	Cohort study	75.0 ± 3.6	342	45/297	3	Yes	9
Luo	2020	China	RCT	75.64 ± 3.11	42	9/37	12	Yes	Low
Martikos	2018	Italy	Cohort study	71.6	85	63/82	24	No	8
Zhang	2016	China	Cohort study	70.20 ± 5.18	160	13/147	12	Yes	8

NOS = Newcastle-Ottawa Scale, RCT = randomized controlled trial, ROB2 = Cochrane Risk of Bias 2 tool.

### 3.3. Risk of bias and study quality

Results of risk of bias assessments for randomized controlled trials and cohort studies are presented in Table 1, respectively. Two randomized controlled trials and 5 cohort studies were considered of good quality and low risk of excursions.

### 3.4. Effect of VA on new vertebral fractures following single-level OVCFs

**Figure 2.**
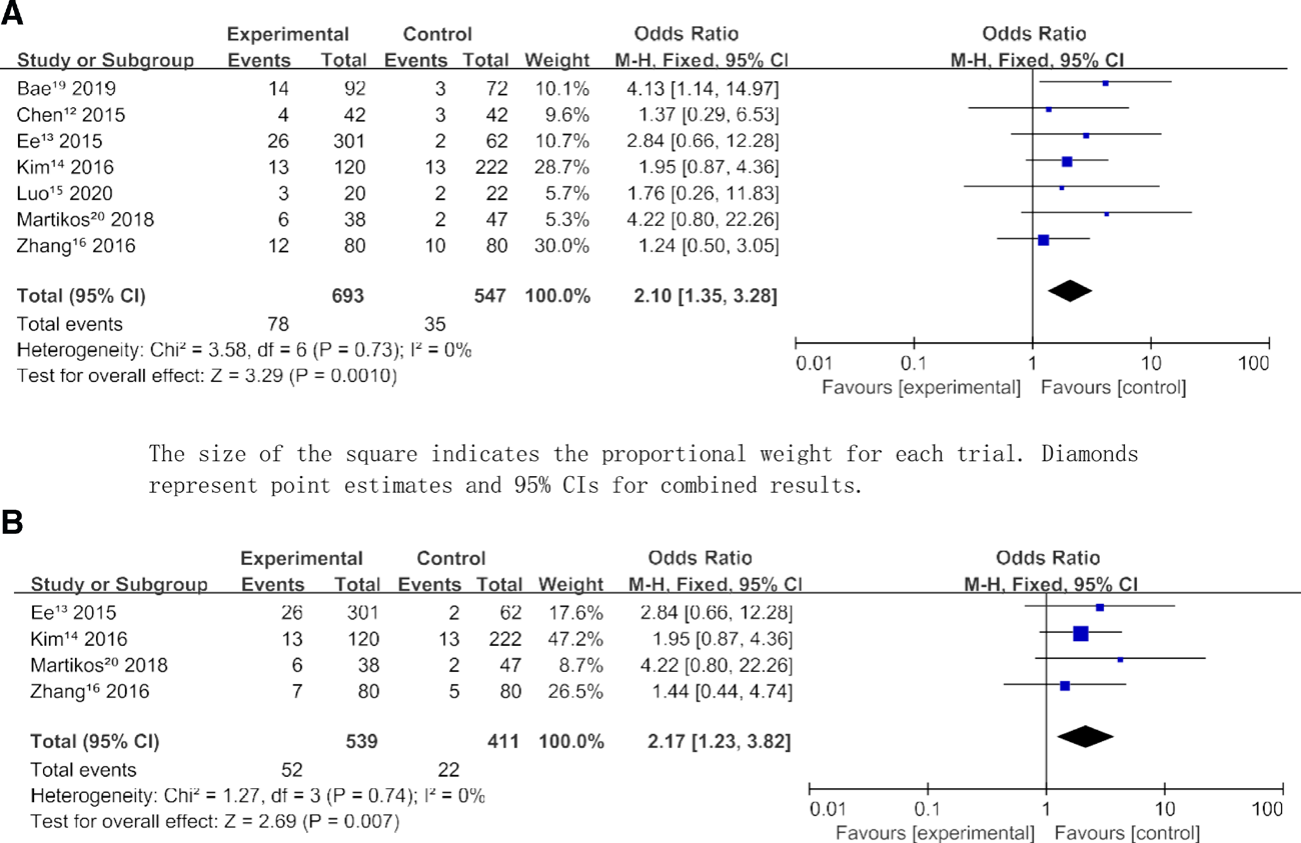
(A) New vertebral fracture. The size of the square indicates the proportional weight for each trial. Diamonds represent point estimates and 95% CIs for combined results. (B) Adjacent vertebral fracture.

**Figure 3.**
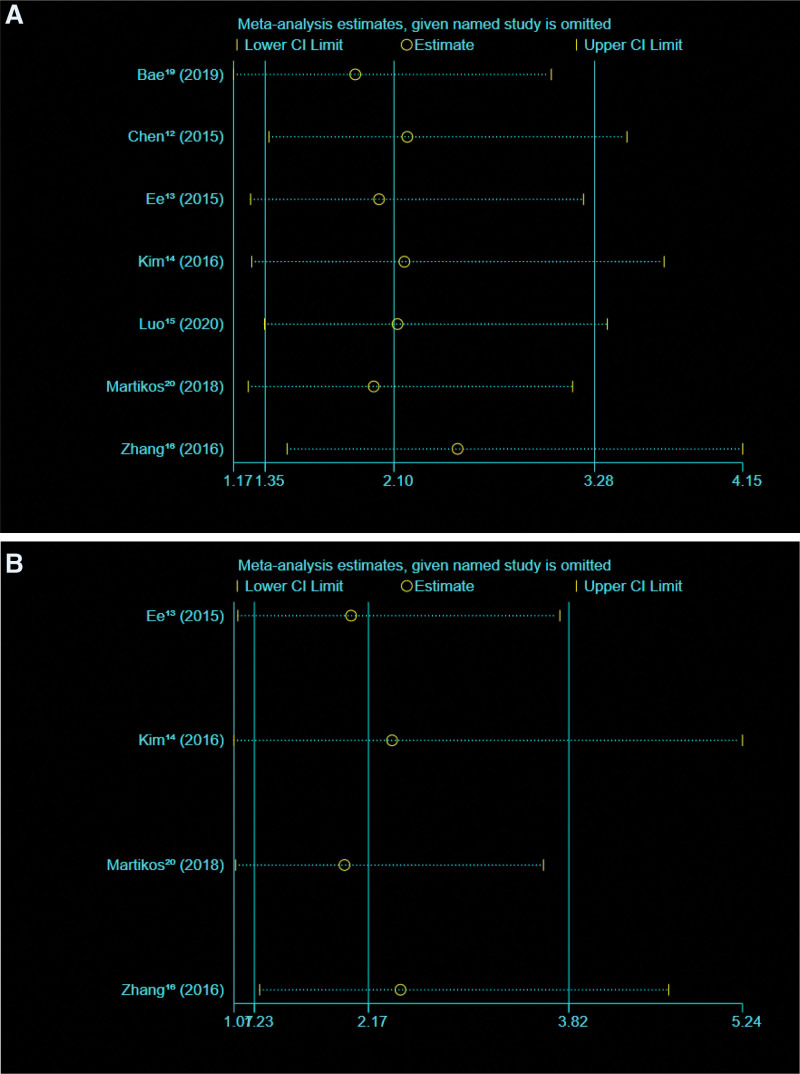
(A) New vertebral fracture. (B) Adjacent vertebral fracture.

A total of 113 new vertebral fractures occurred during the follow-up period in 1240 patients with single-level OVCFs. There were 693 patients in the VA group, 78 (11.26%) of whom developed new vertebral fractures during follow-up. There were 547 patients in the conservative treatment group (CT), 35 (6.40%) of whom developed new vertebral fractures during follow-up. The results of the test showed that VA (OR = 2.10, 95% CI 1.35–3.28, *P* = .001) increased the risk of new vertebral fractures after single-level OVCFs compared with conservative treatment (Figure 2A and B). The heterogeneity test showed Chi^2^ = 3.58, df = 6 (*P* = .73); *I*^2^ = 0%. This suggests little heterogeneity in the included studies. The largest number of study samples was conducted in Singapore by Ee et al, with a total of 363 individuals. Zhang et al performed the largest proportion of study weights in China, reaching 30.0%. Martikos et al had the smallest proportion of study weights in Italy. In terms of sensitivity analysis, we excluded one study at a time and 7 times and observed no significant change in heterogeneity or results. We performed sensitivity analysis on STATA15 software and generated analysis plots (Figure 3A and B).

### 3.5. Subgroups

**Figure 4.**
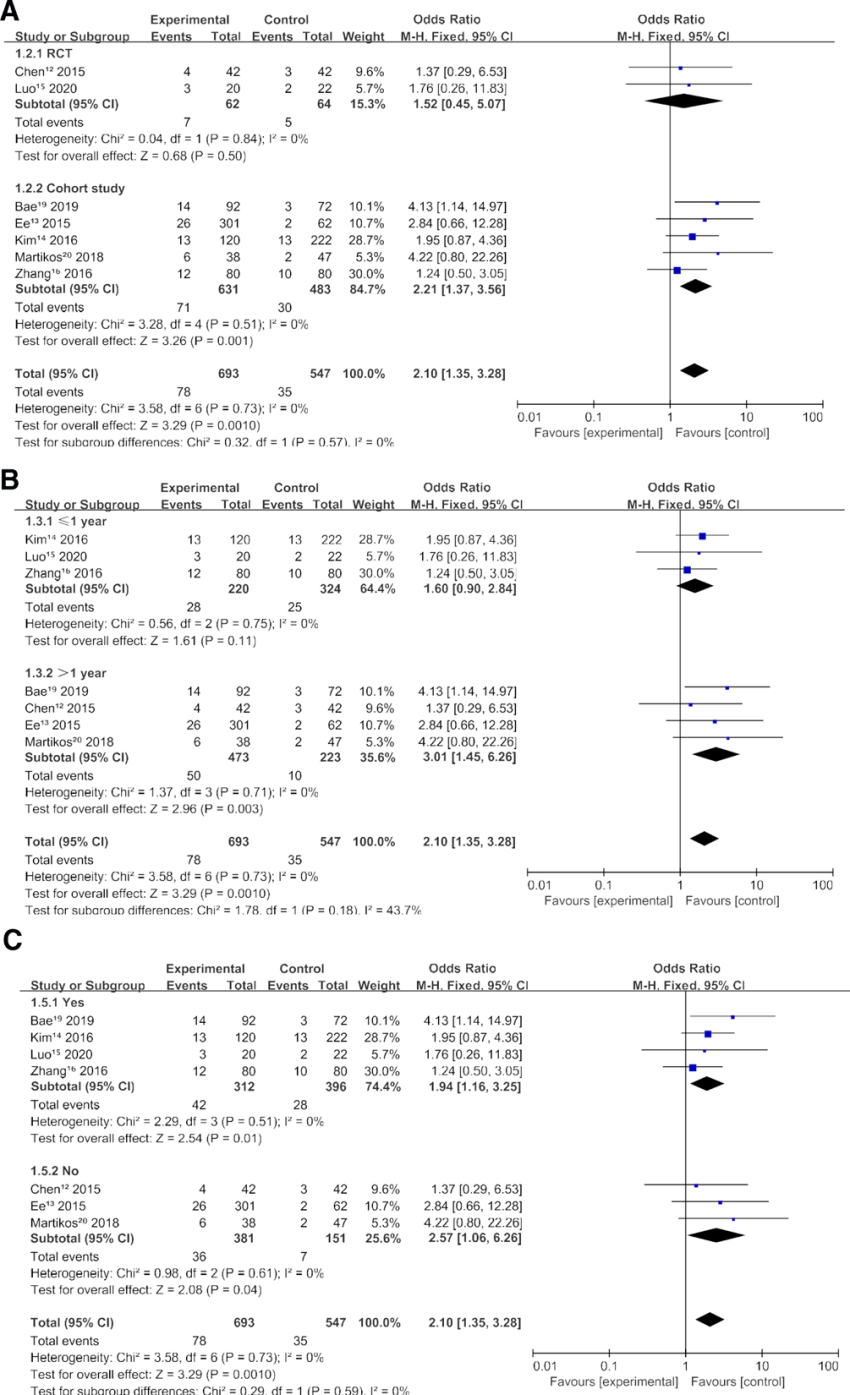
(A) Study design. (B) Follow-up time. (C) Osteoporosis therapy.

We included the study design, whether follow-up was longer than 1 year, whether any patients received osteoporosis treatment as a subgroup, and performed subgroup analysis. Subgroup analysis of study design showed that there was a significant difference in the cohort study group (*P* = .001), there was no significant difference in the randomized controlled trial group (*P* = .50). However, only 2 studies were included in the randomized controlled trial group, and the reliability of the results was low. Subgroup analysis by study design showed no heterogeneity (Chi^2^ = 0.32, df = 1 (*P* = .57), *I*^2^ = 0%). subgroup analysis of follow-up time showed no significant difference between VA and CT groups with follow-up time equal to less than 1 year (*P* = .11), and between VA and CT groups with follow-up time > 1 year (*P* = .003). Subgroup analysis of follow-up time showed moderate heterogeneity (Chi^2^= 1.78, df = 1 (*P* = .18), *I*^2^= 43.7%). Subgroup analysis of osteoporosis treatment showed that there was no significant difference in the treatment group (*P* = .01), and no significant difference in the untreated group (*P* = .04). The results of subgroup analysis of osteoporosis treatment showed no heterogeneity (Chi^2^ = 0.33, df = 1 (*P* = .57), *I*^2^ = 0%) (Fig. 4A–C).

## 4. Discussion

With the rapid growth of the aging population worldwide, the incidence of osteoporosis is increasing year by year.^[1]^ Especially in postmenopausal women, bone mineral density decreases year by year due to a decrease in estrogen secretion in the body.^[23]^ Because of the reduction of bone mineral density, even minor daily external forces are likely to lead to severe fractures.^[24,25]^ The most common OVCFs in osteoporotic fractures can be triggered by bending down this low-weight-bearing daily exercise and has become one of the common diseases afflicting the elderly.

PV and PK in VA have been widely used in the treatment of OVCFs, and many studies have shown that PV/PK can effectively relieve pain and improve function caused by OVCFs.^[3–11]^ Moreover, it has certain advantages in cost-effectiveness.^[26]^ Because the surgical incision is small, the onset speed is fast and the patient acceptance is high, it is accepted by many patients and scholars. However, it seems that too many scholars pay too much attention to postoperative VAS score changes, while there are few reports on the effect of new postoperative vertebral fractures. There is currently a huge debate on this issue. Some scholars believe that VA does not increase the risk of new vertebral fractures after OVCFs.^[9,12–17]^ Because osteoporosis is a degenerative disease and bone mineral density will gradually decrease with age, new vertebral fractures in PV/PK may be inevitable because patients have lower vertebral bone mineral density and the appearance of new vertebral bodies. it seems that too many scholars pay too much attention to postoperative VAS score changes, while there are few reports on the effect of new postoperative vertebral fractures. There is currently a huge debate on this issue. Robert et al^[25]^ investigated the risk of other OVCFs in 2725 postmenopausal women following OVCFs. They concluded that as long as OVCFs occur in postmenopausal women after OVCFs, there is a greater risk of fractures in other vertebral bodies within the next year. Klazen et al^[17]^ concluded that VA did not increase the risk of new vertebral fractures compared with conservative treatment, while there was no difference between adjacent and non-adjacent vertebral fractures. Some scholars believe that VA increases the risk of new vertebral fractures after OVCFs.^[18–22]^ Because bone cement increases the stiffness of the vertebral body, this makes other vertebral bodies more susceptible to fracture. And due to the change in vertebral body height, the mechanical structure of the entire spine has changed, resulting in secondary fractures of other vertebral bodies. Rohlmann et al^[27]^ tried to explain the biomechanically high risk of new vertebral fractures after PV surgery. The results of their finite element analysis indicated that the injected vertebral bodies could rapidly restore vertebral stiffness, thereby changing the stress load of other vertebral bodies and making others vertebral bodies more prone to fracture. Moreover, some surgical complications are also risk factors leading to new vertebral fractures. Berlemann et al^[28]^ showed that cement in the vertebral body increases mechanical stress in other vertebral bodies, causing continued new vertebral fractures in other vertebral bodies. Borensztein et al^[29]^ concluded that the percentage of cement in the vertebral body is associated with new vertebral fractures and kyphotic deformity. Appropriate amounts of cement should be injected, and excess injected cement can lead to excessive vertebral body size and is associated with an increased risk of cement leakage. Rho et al^[30]^ retrospectively reviewed 147 patients who received VA for OVCFs and developed new vertebral fractures postoperatively. They concluded that disc cement leakage is a major risk factor for OVCFs. Having the same view, Liu et al^[31]^ differed in their belief that this result would be more significant at a later stage. Sun et al^[32]^ identified the thoracolumbar junction as an important contributing factor to new vertebral fractures. Because the thoracolumbar junction has greater mobility and is also responsible for the function of weight bearing, it produces great shear force when bending down and is a predilection site for spinal injury.

Many scholars have not studied multilevel OVCFs and single-level OVCFs separately, which may be the reason for the current debate on whether VA increases the risk of new vertebral fractures after OVCFs. Because multilevel OVCFs can have a greater impact on the entire spine than single-level OVCFs, this is even more so after VA treatment. For example, sandwich OVCFs (OVCFs occur in both the upper and lower vertebral bodies of the intact vertebral body), and new vertebral fractures are more likely to occur in the middle intact vertebral body.^[33]^ Deibert et al,^[34]^ in their study of 726 patients who underwent VA surgery, found that all patients with both lumbar and thoracic fractures developed adjacent vertebral fractures, and they concluded that the initial number of vertebral fractures was a contributing factor to adjacent vertebral fractures. Clinically most patients encountered by surgeons involve only a single vertebral body. Therefore, it is necessary to have studies on single-level OVCFs to provide clinicians with some help in selecting treatment options.

This systematic review aimed to reduce the interference of multilevel OVCFs and to investigate the effect of VA for single-level OVCFs on new vertebral fractures. When we first started the search, the preferred study design was a study of randomized controlled trials. However, only 2 articles were obtained in the end. This also illustrates that a large number of high-quality randomized controlled trials are still needed to supplement this aspect at present. So we did not limit the study design. We established that conservative treatment was selected as the control group at the time of the search, which can effectively reduce the heterogeneity of the retrieved studies while improving the level of evidence of the included studies and ensuring the authenticity of the results. We searched for newly published relevant studies at the end of our statistical analysis and started writing papers, and finally, no studies met the inclusion criteria.

Regarding the risk of publication excursion, we produced funnel plots to assess the risk of publication excursion by observing whether it was symmetrical or not. Although we obtained results with a low risk of publication bias, because we included only 7 studies, assessing the risk of publication bias was not necessary.

We also analyzed studies that reported adjacent vertebral fractures to investigate whether VA increases the risk of adjacent vertebral fractures in single-level OVCFs. Trout et al^[35]^ reported that 70% of postoperative fractures occurred in adjacent vertebral bodies. Mechanisms of adjacent vertebral fractures are not infrequently investigated. Among them, stress load transfer in the spine after vertebral augmentation is a more widely accepted mechanism.^[36]^ This is because VA enhances the stiffness of the treated vertebral level and alters the biomechanical properties of the adjacent vertebral body, resulting in increased stress at the level of the adjacent vertebral body. This also supports our view.

Results of subgroup analyses indicated that VA increased the risk of new vertebral fractures following single-level OVCFs at follow-up > 1 year. This may also be why many scholars hold different views. Short-term follow-up of similar outcomes in the VA and CT groups has led some scholars to believe that VA does not increase the risk of new vertebral fractures after OVCFs. However, the difference between the 2 groups became progressively significant with longer follow-ups. Liu et al^[31]^ showed that cement leakage from the disc following VA increases adjacent vertebral fractures, but this effect is barely observed in the short term but increases the risk of adjacent vertebral fractures significantly in the later period. Symptomatic new vertebral fractures in 726 patients treated with PK surgery in the study conducted by Deibert et al^[34]^ are relatively uncommon and may occur long after initial PK surgery. Patients and healthcare workers seem to devote too much attention to postoperative pain relief and ignore the impact of long-term postoperative complications. We think it’s time to turn our attention to long-term postoperative complications.

Some patients in the included studies received bisphosphonates and teriparatide for osteoporosis. We were hesitant at the outset to conduct a subgroup analysis of whether any patients received osteoporosis treatment during the follow-up period. Because in many studies only some people were treated for osteoporosis, not all. Because of this, the final results may not be very credible. However, many studies have demonstrated the importance of bisphosphonates and teriparatide for bone mineral density improvement. So we still performed subgroup analysis. Because the heterogeneity test showed little heterogeneity, we chose a fixed effects model. The results obtained by selecting a random-effects model may be more conservative. We try to change the fixed effects model into a random effects model. Most of the results did not change significantly when the random-effects model was selected. However, the results of the subgroup analysis showed that people who did not receive osteoporosis treatment did not have an increased risk of new vertebral fractures by VA. This is inconsistent with our initial hypothesis, as a large number of high-quality studies have demonstrated that teriparatide or bisphosphonates are effective in increasing bone mineral density. This may also be related to the limitations of the included studies. The study designers need to respect the patient’s choice and not enable all patients to receive the same treatment.

This systematic review has several limitations. The low number of relevant studies. Data from randomized controlled trials and cohort studies were combined for analysis, and these may have resulted in distorted results. The Newcastle-Ottawa Scale has limitations in assessing the internal validity of the study. In addition, some of the included studies did not report baseline characteristics such as comorbidities, specific surgical procedures, cement distribution, and postoperative complications. Because of the limitations of the included studies, may also have influenced our final results. Despite adequate analysis in the discussion, their impact on the results is unclear. The 7 countries studied were mostly Asian countries, so the findings may not be representative of all people.

## 5. Conclusions

VA is associated with an increased risk of new vertebral fractures and adjacent vertebral fractures following single-level OVCFs. With longer follow-ups, new vertebral fractures may be more significant. This study can provide some help for clinical surgeons’ treatment decisions. At the same time, clinical surgeons should not only pay attention to short-term pain after VA but also pay attention to the problem of long-term postoperative complications. Clinicians should carefully select treatment options.

## Author contributions

**Conceptualization:** Zhaoyang Qiu, Peng Wang.

**Data curation:** Zhaoyang Qiu, Peng Wang, Yuqiang Cao, Yang Yu.

**Formal analysis:** Zhaoyang Qiu, Peng Wang, Yuqiang Cao, Yang Yu.

**Investigation:** Zhaoyang Qiu, Yuqiang Cao, Yang Yu.

**Methodology:** Zhaoyang Qiu, Peng Wang, Yuqiang Cao, Yang Yu.

**Project administration:** Peng Wang.

**Resources:** Zhaoyang Qiu, Peng Wang.

**Software:** Zhaoyang Qiu, Peng Wang, Yuqiang Cao, Yang Yu.

**Supervision:** Peng Wang.

**Validation:** Zhaoyang Qiu, Peng Wang, Yuqiang Cao, Yang Yu.

**Writing – original draft:** Zhaoyang Qiu, Peng Wang.

**Writing – review & editing:** Peng Wang, Yang Yu.

## Supplementary Material


