# Supplementary material for: The risk of new vertebral fracture after percutaneous vertebral augmentation in patients suffering from single-level osteoporotic vertebral compression fractures: A meta-analysis and systematic review
Source: Medicine (Baltimore). 2023 Nov 17;102(46):e35749. doi: 10.1097/MD.0000000000035749 (PMC10659685; doi:10.1097/MD.0000000000035749)
Supplement: Supplementary file 1 [file medi-102-e35749-s001.doc]

(((((("Spinal Fractures"[Mesh]) OR (((((((((Fracture, Spinal[Title/Abstract]) OR (Fractures, Spinal[Title/Abstract])) OR (Spinal Fracture[Title/Abstract]))) OR (Hangman Fracture[Title/Abstract])) OR (Fracture, Hangman[Title/Abstract])) OR (Hangman's Fracture[Title/Abstract])) OR (Fracture, Hangman's[Title/Abstract])) OR (Hangmans Fracture[Title/Abstract]))) AND ((("Osteoporosis"[Mesh]) OR (((((((((((((((((((((Osteoporoses[Title/Abstract]) OR (Osteoporosis, Post-Traumatic[Title/Abstract])) OR (Osteoporosis, Post Traumatic[Title/Abstract])) OR (Post-Traumatic Osteoporoses[Title/Abstract])) OR (Post-Traumatic Osteoporosis[Title/Abstract])) OR (Osteoporosis, Senile[Title/Abstract])) OR (Osteoporoses, Senile[Title/Abstract])) OR (Senile Osteoporoses[Title/Abstract])) OR (Osteoporosis, Involutional[Title/Abstract])) OR (Senile Osteoporosis[Title/Abstract])) OR (Osteoporosis, Age-Related[Title/Abstract])) OR (Osteoporosis, Age Related[Title/Abstract])) OR (Bone Loss, Age-Related[Title/Abstract])) OR (Age-Related Bone Loss[Title/Abstract])) OR (Age-Related Bone Losses[Title/Abstract])) OR (Bone Loss, Age Related[Title/Abstract])) OR (Bone Losses, Age-Related[Title/Abstract])) OR (Age-Related Osteoporosis[Title/Abstract])) OR (Age Related Osteoporosis[Title/Abstract])) OR (Age-Related Osteoporoses[Title/Abstract])) OR (Osteoporoses, Age-Related[Title/Abstract]))) OR (("Osteoporotic Fractures"[Mesh]) OR (((Fracture, Osteoporotic[Title/Abstract]) OR (Fractures, Osteoporotic[Title/Abstract])) OR (Osteoporotic Fracture[Title/Abstract]))))) AND (("Fractures, Compression"[Mesh]) OR (((Compression Fracture[Title/Abstract]) OR (Fracture, Compression[Title/Abstract])) OR (Compression Fractures[Title/Abstract])))) AND (("Vertebroplasty"[Mesh]) OR (("Kyphoplasty"[Mesh]) OR ((Balloon Vertebroplasty[Title/Abstract]) OR (Vertebroplasty, Balloon[Title/Abstract]))))) AND ((("Placebos"[Mesh]) OR (Sham Treatment[Title/Abstract])) OR (("Conservative Treatment"[Mesh]) OR (((((((((((Conservative Treatments[Title/Abstract]) OR (Treatment, Conservative[Title/Abstract])) OR (Treatments, Conservative[Title/Abstract])) OR (Conservative Therapy[Title/Abstract])) OR (Conservative Therapies[Title/Abstract])) OR (Therapies, Conservative[Title/Abstract])) OR (Therapy, Conservative[Title/Abstract])) OR (Conservative Management[Title/Abstract])) OR (Conservative Managements[Title/Abstract])) OR (Management, Conservative[Title/Abstract])) OR (Managements, Conservative[Title/Abstract]))))) AND ((("Postoperative Complications"[Mesh]) OR (((Complication, Postoperative[Title/Abstract]) OR (Complications, Postoperative[Title/Abstract])) OR (Postoperative Complication[Title/Abstract]))) OR (("Treatment Outcome"[Mesh]) OR (((((((((((((((((Outcome, Treatment[Title/Abstract])) OR (Patient-Relevant Outcome[Title/Abstract])) OR (Outcome, Patient-Relevant[Title/Abstract])) OR (Outcomes, Patient-Relevant[Title/Abstract])) OR (Patient Relevant Outcome[Title/Abstract])) OR (Patient-Relevant Outcomes[Title/Abstract])) OR (Clinical Effectiveness[Title/Abstract])) OR (Effectiveness, Clinical[Title/Abstract])) OR (Treatment Effectiveness[Title/Abstract])) OR (Effectiveness, Treatment[Title/Abstract])) OR (Rehabilitation Outcome[Title/Abstract])) OR (Outcome, Rehabilitation[Title/Abstract])) OR (Treatment Efficacy[Title/Abstract])) OR (Efficacy, Treatment[Title/Abstract])) OR (Clinical Efficacy[Title/Abstract])) OR (Efficacy, Clinical[Title/Abstract]))))
